# Tuning the structural, morphological, optical, and magnetic properties of hydrothermally synthesized MnSe nanoparticles *via* transition-metal (Cr, Fe, Ni) incorporation

**DOI:** 10.1039/d6ra04668a

**Published:** 2026-07-13

**Authors:** Ali Salmani Nokabadi, Ahmad Yazdani

**Affiliations:** a Tarbiat Modares University Tehran Iran salmaniali.nokabadi@gmail.com

## Abstract

This study presents a strategy for tuning the internal magnetic exchange interactions of manganese selenide (MnSe) nanostructures through selective transition-metal impurity incorporation (Cr, Fe, and Ni). By modifying the local d-electron environment, three distinct structural and magnetic evolution pathways are observed. Iron incorporation promotes a strong phase replacement process, leading to a transition from an antiferromagnetic state to a ferromagnetic response with a saturation magnetization of approximately 11 emu g^−1^. In contrast, chromium and nickel mainly induce phase redistribution and morphological modification while preserving the overall antiferromagnetic character. Significant morphological evolution, including hierarchical and aggregated nanostructures, accompanies these transformations. Despite substantial structural and magnetic changes, the optical band gap remains within a narrow range of 1.68–1.73 eV, indicating that the semiconducting electronic structure is largely preserved. The results demonstrate that the magnetic behavior of MnSe based chalcogenides can be effectively tuned through impurity-dependent exchange interactions while maintaining relatively stable optical properties, providing insight into the design of multifunctional materials for spin caloritronic and spin dependent electronic applications.

## Abstract 2

This study presents a strategy for tuning the internal magnetic exchange interactions of manganese selenide (MnSe) nanostructures through selective transition-metal impurity incorporation (Cr, Fe, and Ni).

## Graphical abstract

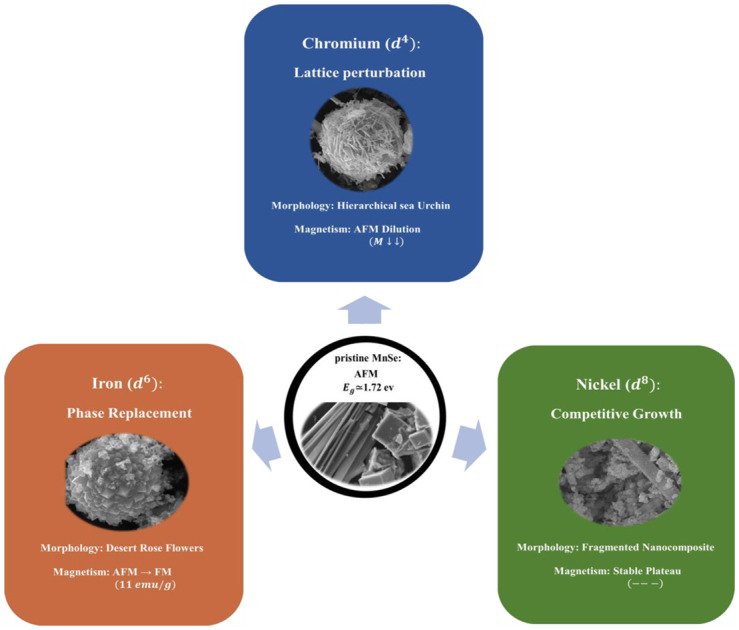

## 1 Introduction

Driven by the growing demand for energy efficient and multifunctional electronic components, research has increasingly focused on materials that exploit the spin degree of freedom. Particularly promising are layered systems in which spintronic and magnetocaloric effects can be coupled, offering new routes for advanced information processing and thermal management applications.^1,2^ Transition-metal chalcogenides stand out in this context, as many members combine magnetic ordering, semiconducting band structures, and rich crystal chemistry. These properties are often mediated by strong spin lattice and magnetoelastic interactions, which inherently link structural and magnetic degrees of freedom.^3–6^ Their layered nature further enables studies of reduced dimensionality and the emergence of unconventional electronic states, such as the superconductivity reported in FeSe under pressure or at interfaces.^2,7–10^ A comparative examination of FeSe and MnSe despite their distinct crystal structures, electronic configurations, and magnetic ground states helps clarify how magnetism and electronic properties evolve across related chalcogenide systems.^11,12^ While FeSe is widely known for its superconductivity and the suppression of long range antiferromagnetic order in low-dimensional forms, MnSe retains robust antiferromagnetism and exhibits pronounced coupling between structural, magnetic, and electronic degrees of freedom.^13,14^ This combination makes MnSe based nanostructures particularly appealing for spin caloritronics, where the coupled transport of spin and heat provides a potential pathway toward integrating thermal management and spin based functionality within a single material system.^15–18^

The research trajectory on MnSe indicates a gradual shift from fundamental studies toward the targeted engineering of its properties. In the early stages, the focus was on characterizing its antiferromagnetic behavior^19^ and the simultaneous investigation of its magnetic, thermal, and electrical properties.^20,21^ In recent decades, attention has shifted toward advanced applications,^22–24^ such as achieving size dependent ferromagnetic like signatures^25^ and evaluating the material's potential for the magnetocaloric effect.^26^ Correspondingly, efforts have concentrated on internal material engineering and the exploration of new states, including reports of pressure induced superconductivity.^11,27^ Nevertheless, the core scientific challenge remains the simultaneous and quantitative control over the material's three main characteristics: structural symmetry, energy gap (*E*_g_), and magnetic ordering, to unlock the full performance of MnSe for spin caloritronics.^28^

As a representative transition-metal chalcogenide (TMC), MnSe crystallizes in the rock salt type cubic phase under the usual synthesis conditions of this work, exhibiting an antiferromagnetic ground state at low temperatures (∼130 K).^29–31^ In our previous work, the capability to tune the properties of MnSe using external stimuli was investigated for the first time: in that study, MnSe nanoparticles were prepared under an applied external magnetic field during synthesis. The results indicated that the magnetic field facilitates a phase transformation from MnSe to MnSe2 by modifying the thermodynamics of nucleation and growth. This process was accompanied by visible changes in lattice strain, morphology, and a noticeable increase in the band gap, thereby providing a new route for modulating the structural, magnetic, and optical properties of MnSe using an externally applied magnetic field.^32^

Building on this earlier work, the present study shifts attention from external magnetic field engineering toward internal magnetic environment engineering. The main objective is to systematically investigate how internally induced perturbations induced by adding transition-metal impurities affect the structural, electronic, and magnetic properties of MnSe. This is achieved by introducing chromium (Cr), iron (Fe), and nickel (Ni) as elemental impurities at various concentrations into the MnSe based precursor system, without assuming deliberate substitution into specific lattice sites. This strategy aims to provide a more intrinsic and tunable route to controlling the magnetic response and phase stability of MnSe by modifying the local magnetic interactions and chemical environment within the material.

The fundamental mechanism governing the magnetic behavior of this system is the quantum mechanical exchange interaction, described by the Heisenberg type Hamiltonian:
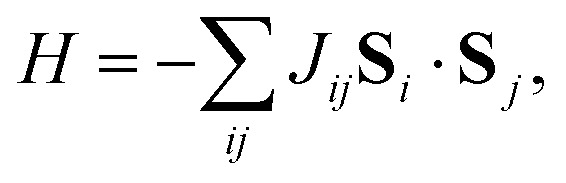
where *J*_*ij*_ is the effective exchange integral and **S**_*i*_, **S**_*j*_ are the spin operators of interacting ions.^33^ The addition of transition-metal ions with different d-electron configurations modifies the local magnetic moments and alters the effective *J*_*ij*_ through changes in orbital overlap, lattice distortions, and crystal field splittings. Chromium (Cr^3+^ analogues, 3d^3^) induce a lower spin multiplicity relative to Mn^2+^ (*S* = 5/2, 3d^5^), perturbing the Mn-centered antiferromagnetic network and introducing new Mn–Cr type exchange pathways. Iron (Fe^2+^, 3d^6^), with an additional 3d electron, can promote local lattice distortions reminiscent of Jahn Teller type effects and shift the balance between competing exchange interactions, potentially leading to distinct magnetic ordering patterns.^34,35^ Nickel (Ni^2+^, 3d^8^), carrying three additional electrons, probes the limits of structural stability and charge balance constraints within the MnSe based matrix, while also influencing the carrier like character near the band edges.

Beyond direct exchange coupling, the impurity induced modifications also influence the local crystal field and band structure, as described by Crystal Field Theory. The electrostatic field from the surrounding selenide (Se^2−^) ligands splits the d-orbitals of both host and impurity ions, and the resulting stabilization energies depend critically on the number and distribution of d-electrons.^34^ For example, asymmetric orbital occupation in Fe^2+^ can yield local lattice distortions that alter the electronic density of states near the Fermi level, thereby affecting conductivity, carrier concentration, and the optical band gap.^35,36^

Accordingly, this study aims to establish a comprehensive structure property relationship that links the electronic configuration of added impurity atoms to the macroscopic magnetic, electronic, and structural responses of the MnSe system. By correlating the impurity driven modifications in internal magnetic environment arising from exchange interactions and crystal field effects with experimental observables such as phase composition, lattice strain, morphology, magnetic moment, and optical gap, this work provides a physically grounded framework for understanding how d-orbital filling governs magnetic transitions, structural stability, and semiconducting behavior in impurity modified MnSe based nanostructures.

## 2 Experimental

All chemicals were purchased from Merck with a purity exceeding 98% and were used as received without further purification. The precursors included manganese nitrate (Mn(NO_3_)_2_), chromium nitrate (Cr(NO_3_)_3_), iron nitrate (Fe(NO_3_)_3_), nickel nitrate (Ni(NO_3_)_2_), selenium powder (Se), and sodium borohydride (NaBH_4_).

**Fig. 1:**
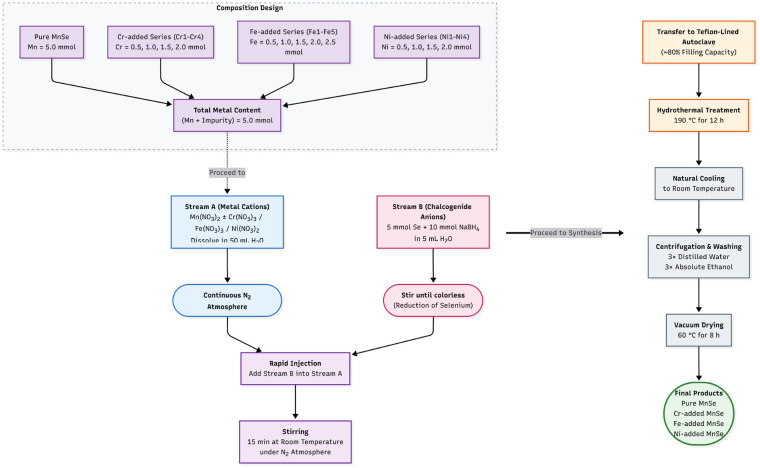
Schematic illustration of the composition design and hydrothermal synthesis procedure for pristine MnSe and impurity-added MnSe samples.

The MnSe nanoparticles, both pure and with added impurity elements, were synthesized using a standard hydrothermal method. The procedure was systematically performed across 14 distinct synthesis stages, with the specific elemental compositions and labeling detailed in Table 1. A comprehensive schematic flowchart illustrating the parallel precursor preparation, composition design, and the step-by-step hydrothermal synthesis process is presented in Fig. 1. For the synthesis of the basic compound, 5 mmol of manganese nitrate was dissolved in 50 mL of water under a nitrogen atmosphere to prevent oxidation. In parallel, 5 mmol of selenium powder was added to a separate solution containing 10 mmol of sodium borohydride in 5 mL of water. This mixture was stirred until a distinct color change from black to colorless was observed, indicating the partial reduction of elemental selenium and formation of a reactive selenide species in solution. The freshly prepared precursor solution was then promptly introduced into the manganese nitrate solution, and the resulting mixture was stirred continuously for 15 minutes to ensure homogeneity and promote precursor mixing.

**Table 1:** Precursor compositions for hydrothermal synthesis of pure MnSe and samples with added elemental impurities

Sample	Mn (mmol)	Cr/Fe/Ni (mmol)	Se (mmol)	NaBH_4_ (mmol)
Pure MnSe	5.0	—	5.0	10.0
Cr1	4.5	0.5 (Cr)	5.0	10.0
Cr2	4.0	1.0 (Cr)	5.0	10.0
Cr3	3.5	1.5 (Cr)	5.0	10.0
Cr4	3.0	2.0 (Cr)	5.0	10.0
Fe1	4.5	0.5 (Fe)	5.0	10.0
Fe2	4.0	1.0 (Fe)	5.0	10.0
Fe3	3.5	1.5 (Fe)	5.0	10.0
Fe4	3.0	2.0 (Fe)	5.0	10.0
Fe5	2.5	2.5 (Fe)	5.0	10.0
Ni1	4.5	0.5 (Ni)	5.0	10.0
Ni2	4.0	1.0 (Ni)	5.0	10.0
Ni3	3.5	1.5 (Ni)	5.0	10.0
Ni4	3.0	2.0 (Ni)	5.0	10.0

For the samples with added impurity elements, a similar procedure was followed, but with part of the manganese nitrate in the precursor mixture replaced by the respective transition-metal nitrates. For these compositions, the molar amount of the host precursor, manganese nitrate (Mn(NO_3_)_2_), was systematically reduced and partially replaced with the corresponding amounts of chromium nitrate (Cr(NO_3_)_3_), iron nitrate (Fe(NO_3_)_3_), or nickel nitrate (Ni(NO_3_)_2_). A critical constraint for all syntheses was maintaining the total metal salt content (Mn + impurity) constant at 5 mmol. The concentrations of the impurity elements were systematically varied to probe the compositional influence on material properties: chromium added samples (Cr-1 to Cr-4): four stages were prepared using 0.5 mmol, 1 mmol, 1.5 mmol, and 2 mmol of chromium nitrate, respectively, with the corresponding molar amount of manganese nitrate reduced so that the total metal salt content remained constant at 5 mmol. Iron added samples (Fe-1 to Fe-5): five stages were synthesized using 0.5 mmol, 1 mmol, 1.5 mmol, 2 mmol, and 2.5 mmol of iron nitrate, respectively, under the same precursor constraint. Nickel added samples (Ni-1 to Ni-4): four stages, mirroring the concentration range of the chromium series, were prepared using 0.5 mmol, 1 mmol, 1.5 mmol, and 2 mmol of nickel nitrate, again with the corresponding reduction in manganese nitrate.

After the initial mixing and stirring, each resulting solution was sealed in a Teflon-lined stainless steel autoclave and heated to 190 °C for 12 hours. Finally, the synthesized samples were washed three times with distilled water and ethanol, with centrifugation performed after each washing step, and subsequently dried at 60 °C for 8 h before further physicochemical characterization.

The dimensions and purity of the resulting materials were assessed utilizing an X-ray diffractometer (XRD) with CuKα radiation (*λ* = 1.54 Å), manufactured by Philips under the X'Pert MPD model. The morphologies of the materials were investigated using a field emission scanning electron microscope (FE-SEM), namely a Hitachi S-4160. Diffuse reflectance spectroscopy (DRS) profiles were recorded with a spectrophotometer spanning the 200–1000 nm range. Magnetic hysteresis loops of the specimens were measured using a vibrating sample magnetometer (VSM) from MDK-magnetics.

## 3 Results

### 3.1 Structural evolution and comparative phase dynamics of impurity modified MnSe

**Fig. 2:**
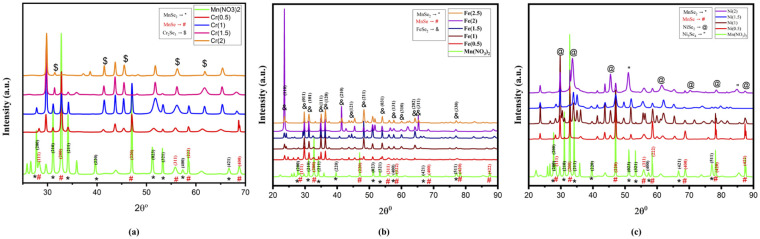
X-ray diffraction patterns of MnSe nanostructures with added impurities: (a) Cr added, (b) Fe added, and (c) Ni added series, illustrating impurity dependent phase evolution and competitive crystallization behavior.

**Fig. 3:**
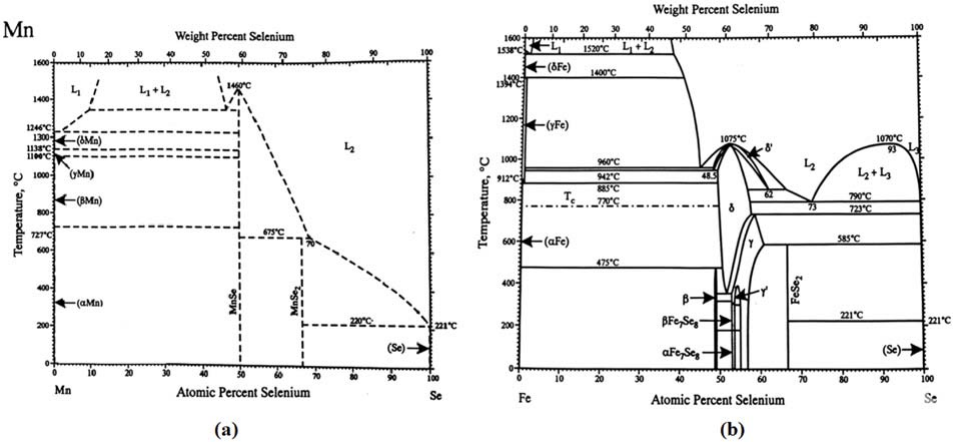
(a) Mn–Se and (b) Fe–Se phase diagrams illustrating the thermodynamic basis for observed phase stabilities.

The X-ray diffraction (XRD) patterns reveal a systematic, element specific evolution in phase composition and lattice parameters as a function of the added transition-metal impurity concentration (Fig. 2). The baseline material synthesized without added impurities consists of a two phase composite of cubic manganese selenide (MnSe) and cubic manganese diselenide (MnSe_2_), with lattice constants of 5.4620 Å and 6.4170 Å (which correspond to JCPDS card numbers 00-011-0683 and 01-073-1525), respectively.^37^ As suggested by the Mn–Se phase diagram (Fig. 3a), the coexistence of these phases is consistent with the thermodynamic behavior of the Mn–Se system,^38^ although the final phase distribution may also be influenced by kinetic effects during hydrothermal synthesis. The introduction of chromium, iron, and nickel leads to distinct changes in the observed phase assemblage, reflecting different chemical affinities and growth pathways for each added element.^39^

In the chromium added series, the XRD response is initially characterized by a slight shift in the MnSe reflections, followed by the appearance of chromium rich phases (Fig. 2a). At 1 mmol Cr, a measurable contraction of the apparent MnSe lattice is observed, where the lattice constant decreases to approximately 5.4400 Å. This peak shift suggests a possible incorporation of chromium into the MnSe lattice or a reduction in the average lattice spacing, consistent with the smaller ionic size of Cr compared with Mn.^40,41^ As the chromium content increases to 1.5 mmol, the phase composition becomes more complex, with coexistence of MnSe, MnSe_2_, monoclinic Cr_2_Se_3_, hexagonal CrSe, and elemental selenium. At the highest chromium content (2 mmol), the contribution of MnSe to the diffraction pattern is strongly reduced, and the dominant phase becomes rhombohedral Cr_2_Se_3_. This trend is consistent with the formation of chromium rich selenides under the present synthesis conditions.

The iron added series shows a more rapid phase replacement than the chromium added series (Fig. 2b). Even at the lowest iron content of 0.5 mmol, the XRD pattern indicates the early formation of orthorhombic iron diselenide (FeSe_2_) alongside other selenium containing phases. Unlike the chromium added samples, which show only a modest shift in the MnSe reflections at low concentration, the iron added samples evolve quickly toward an iron selenide rich phase assemblage. By 2.0 mmol Fe, the manganese-based phases are no longer detectable within the XRD pattern, leaving a composite dominated by orthorhombic FeSe_2_ and elemental selenium.^42^ The Mn–Se (Fig. 3a) and Fe–Se (Fig. 3b) phase relations suggest that iron selenides are favored under these synthesis conditions, but this conclusion should be understood as a phase selection interpretation from XRD rather than a direct thermodynamic measurement. The preferential formation of iron selenide phases indicates that selenium binds readily with iron in the reaction environment, reducing the fraction of manganese containing crystalline products.

The nickel added series exhibits a non-linear phase evolution distinct from both the chromium and iron added samples (Fig. 2c). At 0.5 mmol Ni, cubic MnSe remains the dominant phase, while minor hexagonal NiSe and cubic Ni_3_Se_4_ begin to appear as secondary phases. At 1.5 mmol Ni, the MnSe reflections become very weak and orthorhombic NiSe_2_ becomes more prominent, indicating a shift toward nickel rich selenide formation. At the highest nickel content of 2 mmol, a noticeable re-appearance of the cubic MnSe phase is observed, with reflections near 32.8°, 47.1°, and 55.9°. This suggests that the final phase composition is sensitive to the Mn : Ni precursor ratio and to hydrothermal reaction kinetics. Such behavior is consistent with competition among MnSe and several nickel selenides whose relative stability may be similar under the synthesis conditions.

A comparative analysis of the three systems shows that the added impurity elements influence structural stability and phase selection in different ways, consistent with their distinct electronic configurations and chemical behavior. Chromium addition initially causes a small shift in the MnSe reflections but later leads to chromium rich phase segregation. Iron addition promotes rapid replacement of Mn-containing phases by iron rich selenides, while nickel addition produces a more complex and non-monotonic phase evolution in which no single phase dominates throughout the full composition range. These differences may arise from the interplay of crystal chemistry, lattice mismatch, and phase competition during hydrothermal growth. For example, the 3d electronic structure of iron may favor the formation of stable iron selenide phases under the present conditions. Overall, the XRD results show that the structural identity of MnSe can be modified by adding different impurity elements, leading to three distinct responses: limited lattice perturbation in the chromium added series, complete phase replacement in the iron added series, and non-linear competitive crystallization in the nickel added series.

To provide insight into the structural consequences of impurity addition, the average apparent crystallite size (*D*) was estimated using the Scherrer equation for all identified phases.^41^ To address the multiphase nature of the samples, the Scherrer equation was applied independently to each identified phase using well-resolved, phase-specific diffraction peaks to minimize peak-overlap effects. Because microstrain, which also contributes to peak broadening, is present in the investigated samples and is reported separately in Table 2, the resulting values are referred to as average apparent crystallite sizes. Therefore, these values should be regarded as semi-quantitative indicators of relative grain-growth behavior rather than precise absolute crystallite dimensions. Nevertheless, they provide a physically meaningful basis for comparing structural evolution across the different impurity-added series. These values, summarized in Table 2, reflect the physical impact of impurity-induced perturbations on the MnSe host lattice. The variation in crystallite size provides quantitative information on the effect of impurity addition on crystal growth during hydrothermal synthesis. The reduction in MnSe crystallite size, from approximately 431 Å in the pure sample to about 155 Å for Ni-1.5 mmol, is consistent with the added impurity elements acting as grain-growth inhibitors. At the nanoscale, smaller crystallites have a larger surface to volume ratio, which can increase the influence of surface related strain and defects.

**Table 2:** Crystallite size and lattice strain of synthesized MnSe samples with transition metal impurities calculated *via* Scherrer equation

Series	Conc.	Phase	Crys. size (Å)	Strain (%)
Pure	0.0 mmol	MnSe	431.15	0.259
		MnSe_2_	450.00	0.326
Cr	0.5 mmol	MnSe	223.57	0.443
		MnSe_2_	449.75	0.254
		Cr_2_Se_3_	319.67	0.468
	1.0 mmol	MnSe	392.40	0.194
		MnSe_2_	342.30	0.259
		Cr_2_Se_3_	308.67	0.347
	1.5 mmol	MnSe	362.00	0.257
		MnSe_2_	216.00	0.349
		Cr_2_Se_3_	164.00	0.569
	2.0 mmol	Cr_2_Se_3_	213.75	0.407
Fe	0.5 mmol	MnSe	235.58	0.375
		MnSe_2_	293.13	0.358
		FeSe_2_	342.50	0.269
	1.0 mmol	MnSe	289.00	0.379
		MnSe_2_	178.40	0.659
		FeSe_2_	304.81	0.277
	1.5 mmol	MnSe	265.33	0.419
		FeSe_2_	394.22	0.224
	2.0 mmol	FeSe_2_	288.13	0.288
	2.5 mmol	FeSe_2_	290.10	0.295
Ni	0.5 mmol	MnSe	446.50	0.280
		Ni_3_Se_4_	229.83	0.491
		NiSe	329.50	0.323
	1.0 mmol	MnSe	267.71	0.327
		MnSe_2_	260.60	0.250
		Ni_3_Se_4_	263.00	0.407
		NiSe_2_	202.55	0.499
	1.5 mmol	MnSe	155.20	0.614
		NiSe_2_	166.15	0.603
	2.0 mmol	MnSe	273.29	0.343
		Ni_3_Se_4_	138.00	0.593
		NiSe_2_	188.29	0.500

The impurity related microstrain also shows distinct trends across the three series (Table 2). The chromium added series shows increasing strain in Cr_2_Se_3_, reaching a maximum of 0.569% at 1.5 mmol Cr, which is consistent with lattice mismatch and multiphase coexistence. The iron-added series shows high strain in MnSe_2_, reaching 0.659% at 1 mmol Fe, which may reflect selenium deficient local environments before suppression of the manganese rich phases. The nickel added series exhibits a non-monotonic strain profile, with a peak of 0.614% followed by relaxation, which may reflect competition among nickel rich phases and strain minimization during growth.

### 3.2 Integrated morphological evolution and comparative growth dynamics

**Fig. 4:**
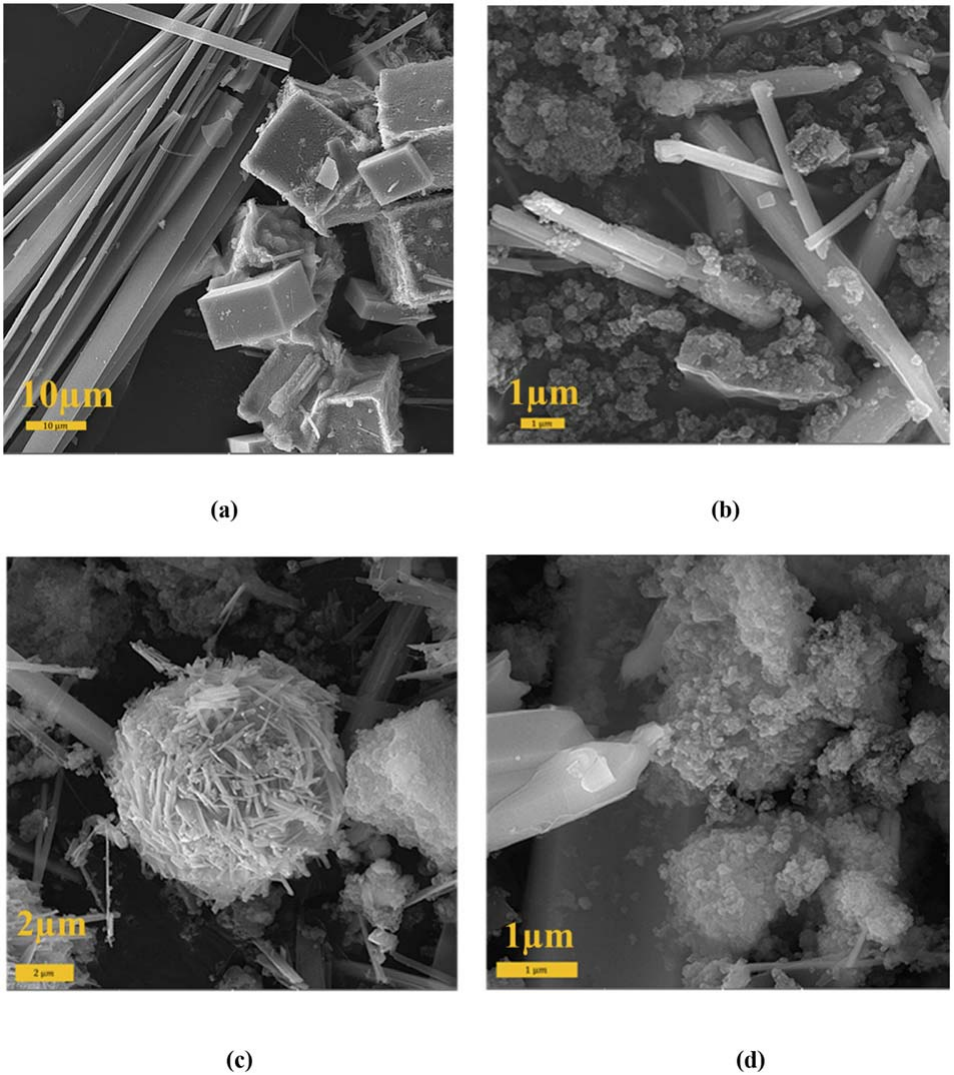
FE-SEM images of pristine and Cr impurity added MnSe nanostructures: (a) baseline bimodal morphology (MnSe nanorods, MnSe_2_ cubes), and evolution of the Cr series from (b) irregular nanoparticle aggregates (1.0 mmol), (c) sea urchin like hierarchical structures (1.5 mmol), to (d) diffusion limited dense aggregates (2.0 mmol).

The morphology of the synthesized materials changes noticeably as the amount of added transition-metal impurity is varied. In the pristine sample, the manganese selenide system exhibits a bimodal distribution of crystal habits, consisting of elongated MnSe nanorods and cubic MnSe_2_ microstructures (Fig. 4a). This morphology suggests that the sample contains two distinct growth behaviors, with one phase forming rod like structures (Fig. S1a, SI) and the other forming more compact cubic particles (Fig. S1b, SI). The sharp edges and relatively smooth surfaces of these microcrystals indicate a high degree of crystallinity and provide a reference state for the impurity added samples.

The introduction of chromium (Cr) leads to a concentration dependent change from ordered microstructures toward more irregular and hierarchical morphologies. At 1.0 mmol Cr, the microstructure becomes less uniform, and irregular nanoparticle aggregates begin to appear (Fig. 4b). As the chromium content reaches 1.5 mmol Cr (15.87% Mn, 19.46% Se, 24.37% Cr) (Fig. S2a, SI), the morphology evolves into a hierarchical, sea urchin-like structure, in which rod-like building blocks are assembled into spherical superstructures (Fig. 4c). This change is consistent with a growth process in which particle aggregation and anisotropic assembly become more pronounced. At 2.0 mmol Cr (23.85% Mn, 58.53% Se, 17.61% Cr) (Fig. S2b, SI), the morphology becomes dominated by dense nanoparticle aggregates, and the original ordered microcrystals are no longer prominent (Fig. 4d).

**Fig. 5:**
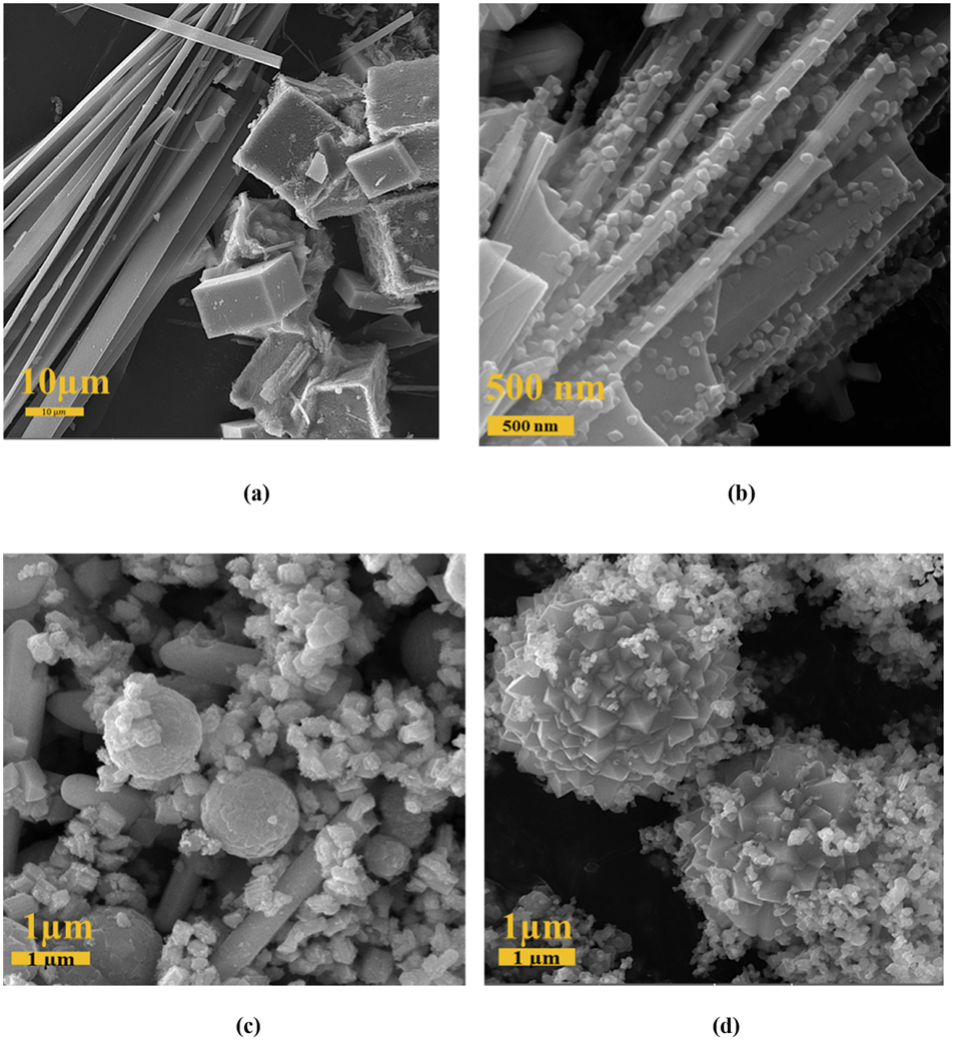
Morphological evolution of MnSe following the addition of iron impurity: (a) pristine state, (b) heterogeneous nucleation on nanorod templates (1.5 mmol Fe), (c) intermediate thickened rod formation (2.0 mmol Fe), and (d) hierarchical FeSe_2_ desert rose architecture (2.5 mmol Fe).

The iron (Fe) series exhibits a more pronounced morphological transformation compared with the chromium added samples. At intermediate iron concentrations, the rod like structures remain observable but become thicker and less uniform, indicating progressive disruption of the original anisotropic growth mechanism. In particular, the 1.5 mmol Fe sample (33.55% Mn, 6.76% Fe, 27.89% Se) (Fig. S3a, SI) retains MnSe nanorods as the primary structural framework, accompanied by the emergence of additional particulate features on their surfaces (Fig. 5b). Upon increasing the iron content to 2.0 mmol Fe (31.10% Mn, 24.32% Fe, 44.59% Se) (Fig. S3b and c, SI), the morphology evolves into a more compact and irregular architecture, suggesting intensified competition between different crystallization and growth pathways (Fig. 5c). At the highest iron concentration (2.5 mmol Fe), the sample develops a flower like (desert rose like) morphology composed of self-assembled crystalline subunits (Fig. 5d). The formation of these hierarchical microstructures reflects a strong tendency toward self assembly and anisotropic crystal growth under iron rich synthesis conditions (Fig. S3d, SI).

**Fig. 6:**
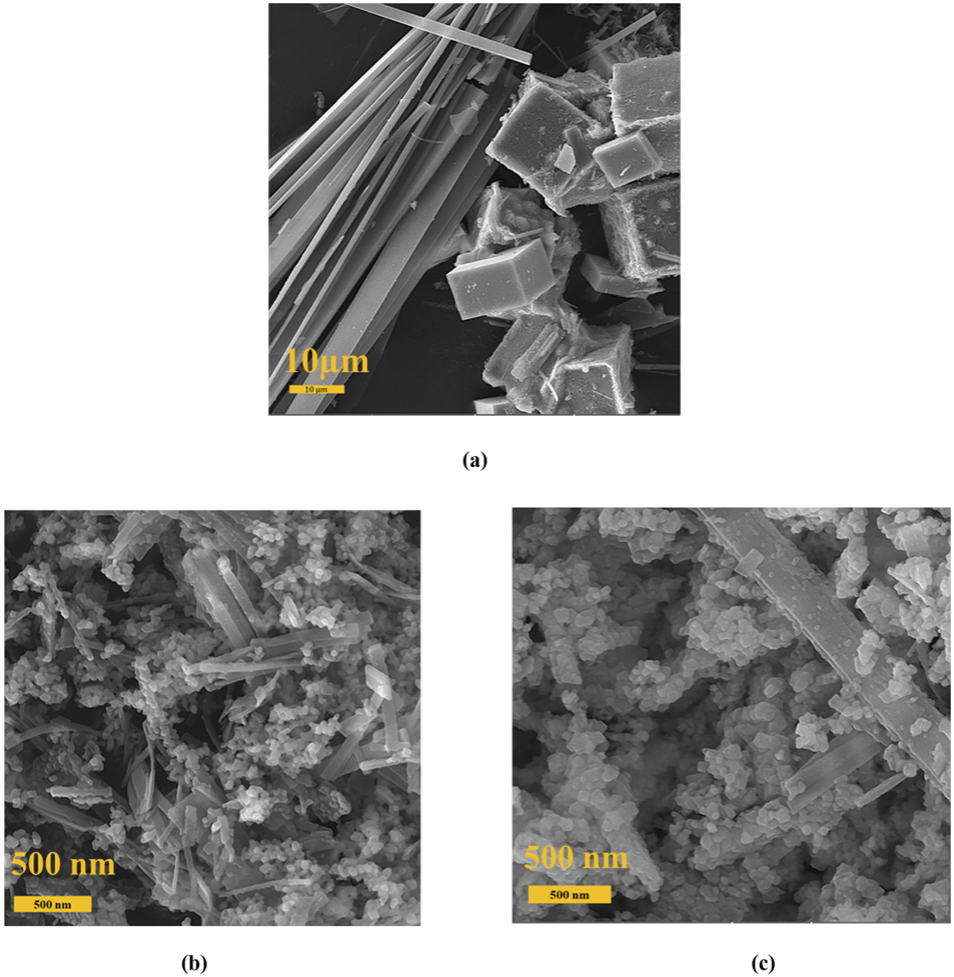
FE-SEM micrographs of MnSe nanostructures with added Ni impurity: (a) pristine baseline, (b) rod fragmentation and growth arrest (1.5 mmol Ni), and (c) multiphase, mixed-phase morphology driven by competitive crystallization (2.0 mmol Ni).

The nickel (Ni) series follows a non-linear morphological pathway. At 1.5 mmol Ni, the well defined rods become fragmented into shorter segments embedded in a nanoparticle matrix, indicating disruption of the original anisotropic growth process (Fig. 6b). By 2.0 mmol Ni (21.44% Mn, 18.14% Ni, 60.42% Se) (Fig. S4, SI), the morphology evolves into a mixed multiphase product with coexisting rod like and particulate features, consistent with competitive growth of multiple phases (Fig. 6c). This behavior suggests that nickel addition does not produce a single uniform morphology, but instead promotes a heterogeneous structure formed through simultaneous crystallization pathways.

Comparatively, these three series illustrate different physical mechanisms of internal environment engineering. Chromium provides a master variable for tuning the system from micro-scale rods to hierarchical spheres through surface energy modulation. Iron drives an aggressive phase replacement that redefines the material's physical form into complex microspheres. Nickel, in contrast, leads to a disordered, high surface area mixed morphology arising from competitive crystallization. Despite these different pathways, all three series result in a dramatic increase in the surface to volume ratio as they transition toward nanostructured forms.

Ultimately, the evolution from ordered microcrystals to complex hierarchical superstructures and mixed-phase nanostructures is of critical functional importance. The high density of active sites and magnetic interfaces generated in these nanostructured materials can influence surface-dependent phenomena such as catalytic activity and interfacial magnetic exchange interactions. By correlating the specific transition-metal impurity configuration with the resulting morphological habit whether it be the sea urchin like structures in the Cr added series, the flower like microspheres in the Fe added series, or the mixed phase morphologies in the Ni-added series this study demonstrates how the internal environment engineering through elemental impurity addition can be used to tune the macroscopic physical behavior of MnSe based materials.

### 3.3 Integrated magnetic analysis and comparative exchange dynamics

**Fig. 7:**
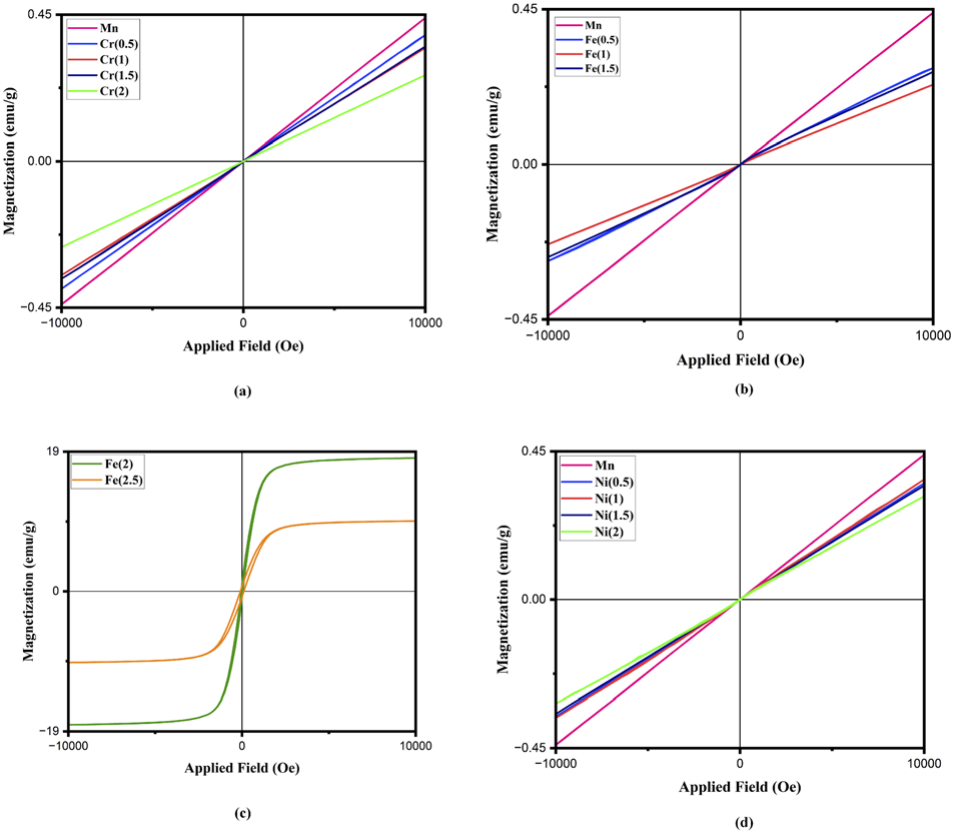
Comparative magnetic analysis of impurity-added MnSe systems: (a) Cr-induced antiferromagnetic dilution, (b) initial magnetic dilution in Fe-containing samples, (c) Fe-driven transition toward robust ferromagnetism, and (d) Ni-stabilized magnetic plateau behavior.

Magnetization measurements of the synthesized nanostructures reveal that the magnetic response of the MnSe system is highly sensitive to the type and concentration of the added transition-metal impurities. In the pristine sample, the MnSe based material exhibits a weak, linear, and nearly non-hysteretic field dependence, with a maximum induced magnetization of 0.44 ± 0.01 emu g^−1^ (Fig. 7a). This behavior is consistent with an antiferromagnetic (AFM) ground state arising from the antiparallel or nearly antiparallel spin alignment of high spin Mn^2+^ ions (3d^5^, *S* = 5/2), coupled through superexchange interactions along Mn–Se–Mn pathways.^43^ To provide a comprehensive quantitative comparison of these magnetic transitions, the key magnetic parameters extracted from the hysteresis loops—including saturation magnetization (*M*_s_), remanent magnetization (*M*_r_), and coercivity (*H*_c_)—for all pure and impurity-added structures are systematically summarized in Table 3.

**Table 3:** Quantitative summary of the magnetic properties, including saturation magnetization (*M*_s_), remanent magnetization (*M*_r_), and coercivity (*H*_c_), extracted from VSM measurements at room temperature

Sample	Impurity conc.	*M*_s_ (emu g^−1^)	*M*_r_ (emu g^−1^)	*H*_c_ (Oe)
MnSe	0.0 mmol	0.440	0.0007	17.96
Cr1	0.5 mmol	0.392	0.0002	5.63
Cr2	1.0 mmol	0.350	0.0001	3.20
Cr3	1.5 mmol	0.361	0.0014	33.83
Cr4	2.0 mmol	0.264	0.0002	7.99
Fe1	0.5 mmol	0.281	0.0007	19.34
Fe2	1.0 mmol	0.232	0.0006	18.47
Fe3	1.5 mmol	0.269	0.0006	14.14
Fe4	2.0 mmol	18.131	0.9713	46.70
Fe5	2.5 mmol	9.682	0.8482	98.81
Ni1	0.5 mmol	0.354	0.0005	13.42
Ni2	1.0 mmol	0.365	0.0010	22.09
Ni3	1.5 mmol	0.347	0.0007	17.62
Ni4	2.0 mmol	0.315	0.0014	34.31

The introduction of chromium (Cr) leads to a progressive reduction in the magnetic moment per unit mass, while the overall magnetic response remains qualitatively similar to that of the pristine sample (Fig. 7a). At 2 mmol Cr, the saturation magnetization decreases to 0.26 ± 0.01 emu g^−1^, indicating magnetic dilution induced by the chromium containing phases. From the perspective of Crystal Field Theory, Cr^3+^ ions (3d^3^, *S* = 3/2) in an octahedral selenide environment adopt a t^3^_2g_e^0^_g_ electronic configuration, which differs significantly from the half filled 3d^5^ configuration of the host Mn^2+^ ions.^44^ This electronic mismatch perturbs the local superexchange pathways, resulting in a weaker macroscopic magnetic response that can be qualitatively interpreted within the Heisenberg exchange framework.

In contrast to the gradual reduction observed in the chromium added series, the iron added samples exhibit a more dramatic evolution in magnetic behavior. At low iron concentrations (*e.g.*, 1.0 mmol Fe), the magnetization continues to decrease, reaching approximately 0.23 ± 0.01 emu g^−1^, consistent with further magnetic dilution (Fig. 7b). Importantly, at these lower concentrations, the material does not exhibit magnetic saturation. This linear, non-saturating M–H profile indicates that the system remains heavily dominated by the antiferromagnetic MnSe matrix, where the external field induces only a slight spin canting of the antiparallel Mn^2+^ sublattices rather than achieving full spin alignment, a behavior characteristic of spin-canted antiferromagnets. However, as the iron content increases beyond 2.0 mmol, the magnetization loop becomes clearly hysteretic, and at 2.5 mmol Fe, the sample exhibits robust ferromagnetic behavior, with a saturation magnetization of 11 ± 1 emu g^−1^ and a well-defined hysteresis loop (Fig. 7c). This transition is consistent with the formation of iron rich selenide phases that favor positive exchange coupling (*J* > 0) and long range spin ordering.

The nickel-added series follows a distinct magnetic trajectory characterized by a stable magnetic moment plateau rather than a monotonic decrease (Fig. 7d). After an initial reduction to 0.35 ± 0.01 emu g^−1^ at 0.5 mmol Ni, the magnetization remains nearly constant up to 1.5 mmol Ni. Similar to the low-concentration iron samples, the nickel-added series does not reach magnetic saturation. This lack of saturation confirms that the Ni impurities do not establish a long-range ferromagnetic network; instead, the response is governed by the robust antiferromagnetic background and localized paramagnetic states. Neither antiferromagnetic nor paramagnetic systems are expected to reach saturation under moderate applied fields, consistent with the observed linear M–H response. This behavior is consistent with the presence of competing magnetic contributions from MnSe and various nickel selenide phases (NiSe_2_ and Ni_3_Se_4_). The Ni^2+^ ion (3d^8^, *S* = 1) in an octahedral selenide field adopts a t^6^_2g_e^2^_g_ electronic configuration that supports a relatively stable, although smaller, spin moment. Furthermore, the coexistence of multiple phases may lead to a quasi equilibrated, non-diluting magnetic response governed by the interplay of several Heisenberg-type exchange interactions.

Overall, comparison of the three impurity series highlights how 3d-orbital filling and the resulting phase composition influence the macroscopic magnetic behavior of the MnSe-based system. Varying the specific concentrations of these transition metals represents a systematic shift from isolated impurity incorporation, which locally perturbs the host lattice, to a regime where secondary metal-selenide phases increasingly influence the macroscopic magnetic response. Chromium addition primarily acts as a magnetic diluent, reducing the effective magnetic moment without fundamentally altering the AFM character. In the Cr-added series, the progressive weakening of the effective exchange interaction suggests an increase in spin fluctuations within the antiferromagnetic background. Such fluctuation-rich antiferromagnetic systems are of interest for spin caloritronic studies, where thermally driven spin currents may in principle be explored. Iron addition drives a strong magnetic phase transition toward a ferromagnetic-like state, consistent with the formation of iron-rich selenide phases. Conversely, the addition of Fe and Ni introduces stronger exchange interactions, thereby suppressing spin fluctuations. In particular, the Fe-added series establishes robust room-temperature ferromagnetism with well-defined coercivity and remanence, providing the magnetic bistability that is a fundamental prerequisite for spintronic memory applications. In contrast, the Ni-added series exhibits a more complex magnetic response governed by competing magnetic interactions and coexisting phases, which may offer opportunities for further investigation of spin-dependent magnetic phenomena. These contrasting magnetic responses motivate future transport, magnetotransport, and spin Seebeck coefficient measurements on these materials. These diverse outcomes ranging from diluted antiferromagnetic behavior to long-range ferromagnetic ordering demonstrate how internal environment engineering through transition-metal impurities can be employed to tailor the magnetic response of MnSe-based chalcogenides for specific next-generation technological applications.

### 3.4 Energy gap

**Fig. 8:**
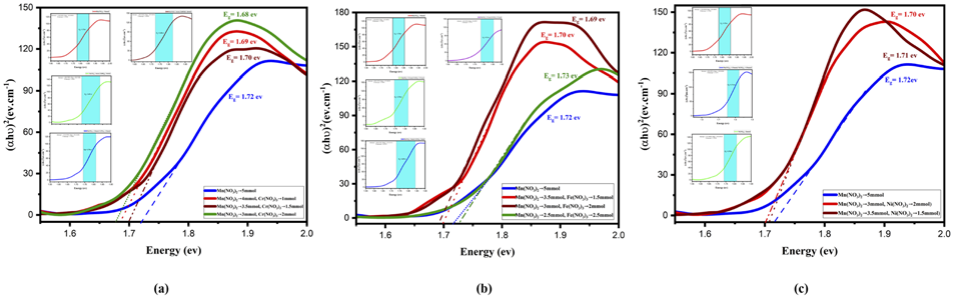
Tauc plots of (*αhν*)^2^*versus* photon energy (*hν*) for the determination of the optical band gap (*E*_g_) of pure and transition-metal-added MnSe samples: (a) Cr-added series, (b) Fe-added series, and (c) Ni-added series (the estimated fitting error for all extrapolated band gap values is approximately ±0.02 eV).

The optical energy gaps (*E*_g_) were estimated by extrapolating the linear portion of the (*αhν*)^2^*versus hν* plots to the energy axis, assuming a direct allowed transition for the dominant absorbing phase. For the pure baseline sample, the energy gap is 1.72 ± 0.02 eV. This calculated value includes a fitting estimated error of approximately ±0.02 eV, derived from the standard deviation of the linear regression applied to the absorption edge tangents. In the chromium-added series (Fig. 8a), the gap decreases slightly and systematically with increasing chromium content, from 1.70 ± 0.02 eV at 1 mmol to 1.69 ± 0.02 eV at 1.5 mmol and 1.68 ± 0.02 eV at 2 mmol. The iron-added series follows a non-linear but similarly narrow range (Fig. 8b), with values of 1.70 ± 0.02 eV at 1.5 mmol, 1.69 ± 0.02 eV at 2 mmol, and a slight increase to 1.73 ± 0.02 eV at the highest concentration (2.5 mmol). The nickel-added series shows the smallest variation (Fig. 8c), with the gap shifting only to 1.71 ± 0.02 eV at 1.5 mmol and 1.70 ± 0.02 eV at 2 mmol.

Despite the pronounced structural changes and phase replacements observed in the XRD analysis—such as the strong transition toward orthorhombic FeSe_2_ in the iron-added series—the optical gap remains remarkably stable. Because the total variation observed across all samples (1.68–1.73 eV) falls largely within the calculated ±0.02 eV fitting uncertainty margin, it can be concluded that the fundamental excitation threshold is practically invariant. This optical stability suggests either that the dominant optical transition is still associated with Mn-containing regions or that the new iron- and nickel-rich phases possess frontier orbital energies closely matching those of the original MnSe-based semiconducting matrix. Even as the long-range symmetry evolves from cubic to orthorhombic or monoclinic, the short-range coordination and p–d hybridization between the metal 3d states and selenium 4p states likely remain the primary determinants of the excitation threshold. Therefore, the effective semiconducting character of the system is robust against the internal environment engineering induced by the added transition-metal impurities.

The persistent optical gap further indicates that no strong band edge shift occurs at the onset of phase replacement, which would be expected if the new phase introduced qualitatively different frontier orbitals. In the iron-added series, even when iron-rich selenides dominate the XRD pattern, the negligible change in *E*_g_ implies that either residual Mn–Se regions still contribute strongly to the optical response or that the iron-rich phases share a similar band structure near the band edges. According to Crystal Field Theory, the high-spin 3d^5^ configuration of Mn^2+^ gives rise to a stable exchange-split electronic structure. The fact that the gap does not collapse or expand significantly outside the error margin suggests that the added d^3^, d^6^, and d^8^ impurities do not create deep-level mid-gap states that would strongly perturb the optical threshold. The introduced impurities may instead integrate into the host electronic manifold through shallow-level or band-edge modifications, without dramatically altering the ligand-to-metal charge transfer (LMCT) energy associated with the selenium anions and the transition-metal cations.

## 4 Conclusions

This study shows that tailoring the internal magnetic environment of MnSe through the addition of transition-metal impurities (Cr, Fe, Ni) enables strong, element specific control over structure, morphology, and magnetism without fundamentally altering the semiconducting character of the system. XRD and morphology analyses reveal three distinct response regimes: limited lattice parameter variation and phase segregation for Cr addition, aggressive phase replacement for Fe, and non-linear competitive crystallization for Ni, driven by differences in ionic size, valence, and selenium affinity. These structural and morphological changes lead to distinct magnetic behaviors, where Cr addition weakens the antiferromagnetic response, Fe drives a transition toward ferromagnetic ordering, and Ni stabilizes an intermediate magnetic plateau controlled by competing Mn and Ni containing phases. Remarkably, despite substantial structural and magnetic reorganization—particularly in the Fe series—the optical band gap remains nearly invariant around *E*_g_ ≈ 1.7 eV, indicating that the band edge electronic states are dominated by Mn–Se-derived p–d hybridization and are resilient to impurity induced perturbations. Together, these results establish a clear structure property function relationship in MnSe, demonstrating that magnetic order can be widely tuned while preserving electronic integrity, a key requirement for spin caloritronic and multifunctional spin based device applications.

## Author contributions

Dr Ahmad Yazdani came up with the idea and oversaw the project. Ali Salmani Nokabadi carried out all synthesis steps and measurements and wrote the manuscript.

## Conflicts of interest

There are no conflicts to declare.

## Supplementary Material

RA-OLF-D6RA04668A-s001

## Data Availability

The datasets generated and/or analysed during the current study are not publicly available because they form part of an ongoing research project. However, the data supporting the findings of this study are available from the corresponding author upon reasonable request. All experimental procedures, characterization data, and analytical methods necessary to evaluate the conclusions of this work are included in the manuscript. Additional information may be provided by the corresponding author if requested by the journal or qualified researchers for academic purposes. Supplementary information (SI): the EDX analysis and elemental mapping results of the samples. See DOI: https://doi.org/10.1039/d6ra04668a.
