# Supplementary material for: Tuning the structural, morphological, optical, and magnetic properties of hydrothermally synthesized MnSe nanoparticles via transition-metal (Cr, Fe, Ni) incorporation
Source: RSC Adv. 2026 Jul 13. Online ahead of print. doi: 10.1039/d6ra04668a (PMC13361203; doi:10.1039/d6ra04668a)
Supplement: RA-OLF-D6RA04668A-s001 [file RA-OLF-D6RA04668A-s001.pdf]

Supplementary Information (SI) for RSC Advances.
This journal is © The Royal Society of Chemistry 2026

Electronic Supplementary Information (ESI)

Tuning the Structural, Morphological, Optical, and Magnetic Properties of Hydrothermally Synthesized MnSe Nanoparticles via Transition-Metal (Cr, Fe, Ni) Incorporation

Ali Salmani Nokabadi and Ahmad Yazdani

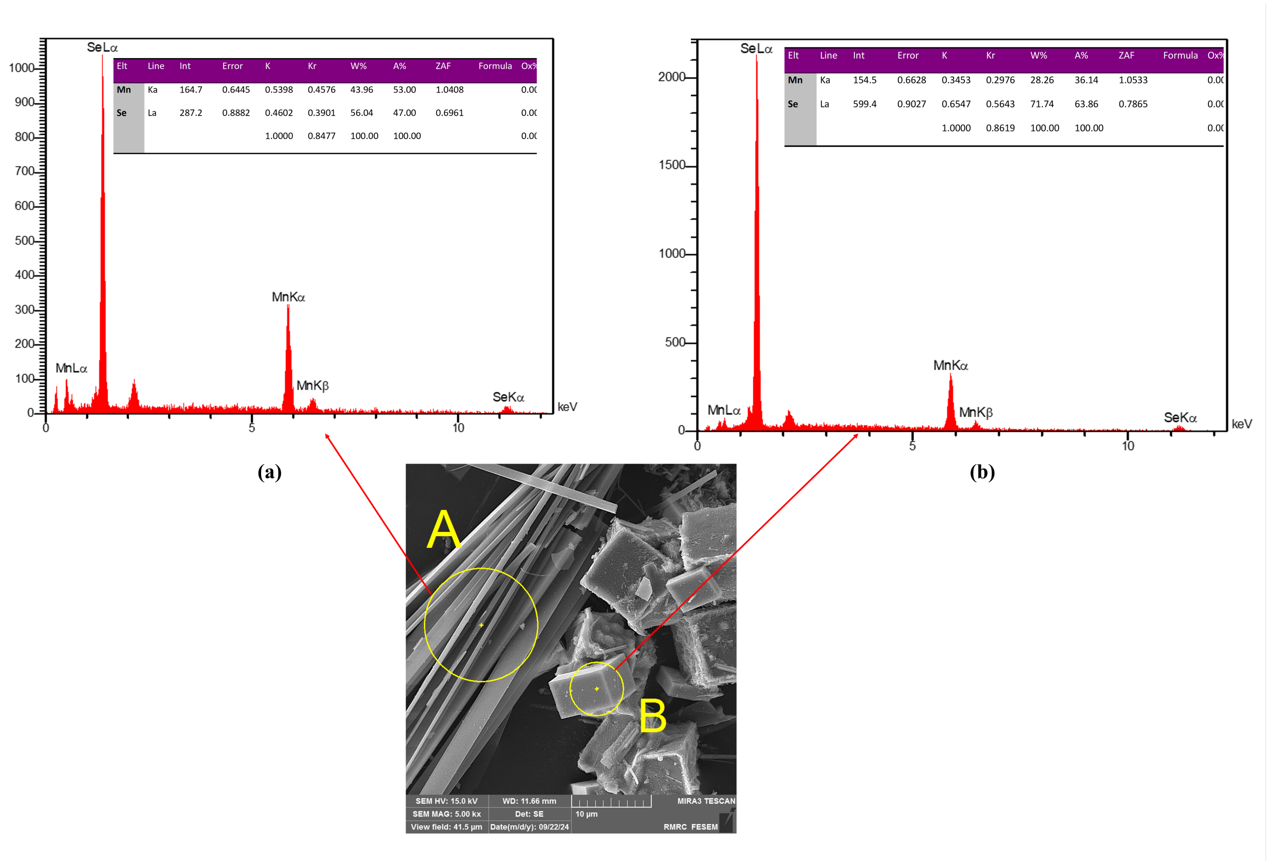

Figure S1: Representative Energy-Dispersive X-ray Spectroscopy (EDS) spectra of the pristine baseline sample, confirming the elemental composition of the coexisting structural phases: (a) the manganese selenide (MnSe) phase and (b) the manganese diselenide ($MnSe_2$) phase.

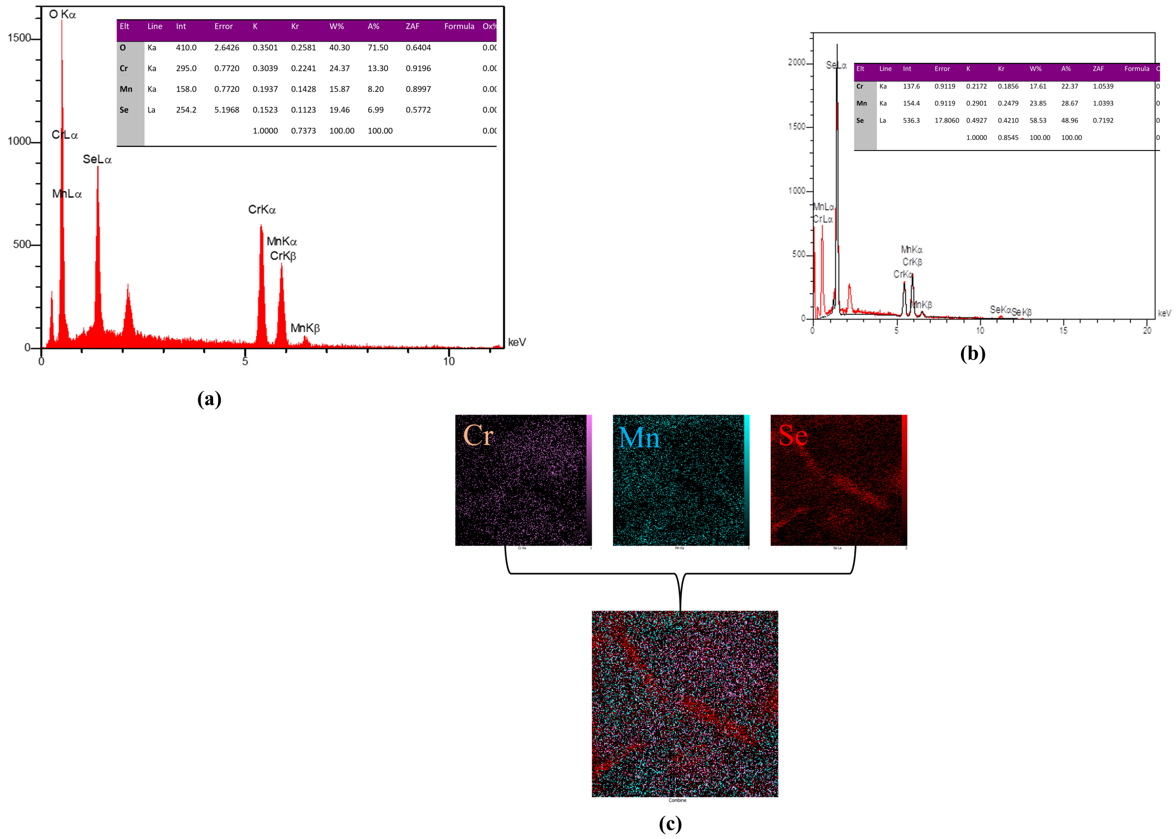

Elt	Line	Int	Error	K	Kr	W%	A%	ZAF	Formula	Ox%
O	Ka	410.0	2.6426	0.3501	0.2581	40.30	71.50	0.6404		0.0[illegible]
Cr	Ka	295.0	0.7720	0.3039	0.2241	24.37	13.30	0.9196		0.0[illegible]
Mn	Ka	158.0	0.7720	0.1937	0.1428	15.87	8.20	0.8997		0.0[illegible]
Se	La	254.2	5.1968	0.1523	0.1123	19.46	6.99	0.5772		0.0[illegible]
				1.0000	0.7373	100.00	100.00			0.0[illegible]

Elt	Line	Int	Error	K	Kr	W%	A%	ZAF	Formula	[illegible]
Cr	Ka	137.6	0.9119	0.2172	0.1856	17.61	22.37	1.0539		0[illegible]
Mn	Ka	154.4	0.9119	0.2901	0.2479	23.85	28.67	1.0393		0[illegible]
Se	La	536.3	17.8060	0.4927	0.4210	58.53	48.96	0.7192		0[illegible]
				1.0000	0.8545	100.00	100.00			0[illegible]

Figure S2: Energy-Dispersive X-ray Spectroscopy (EDS) analysis of the chromium-added MnSe nanostructures: EDS spectra of the samples synthesized with nominal impurity concentrations of (a) 1.5 mmol Cr and (b) 2.0 mmol Cr, confirming the successful incorporation of chromium into the solid phase. (c) Corresponding EDS elemental mapping of the 2.0 mmol Cr-added sample, illustrating the spatial distribution of the constituent elements within the aggregated nanostructures.

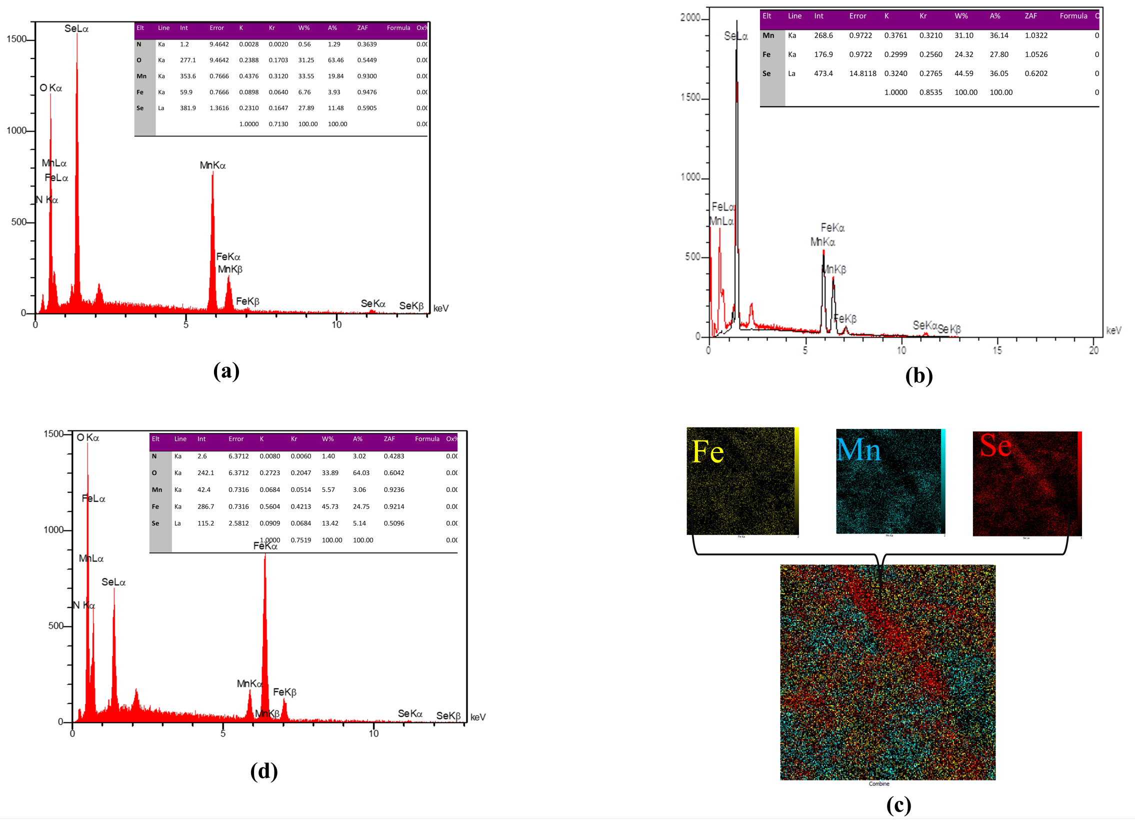

Figure S3: Energy-dispersive X-ray spectroscopy (EDS) analysis of iron-added MnSe nanostructures: EDS spectra of samples synthesized with nominal Fe concentrations of (a) 1.5 mmol and (b) 2.0 mmol; (c) corresponding EDS elemental mapping of the 2.0 mmol Fe-added sample, illustrating the spatial distribution of the constituent elements throughout the synthesized microstructures; and (d) EDS spectrum of the 2.5 mmol Fe-added sample, demonstrating the progressive incorporation of iron and corroborating the phase-transition process toward iron-rich selenide phases.

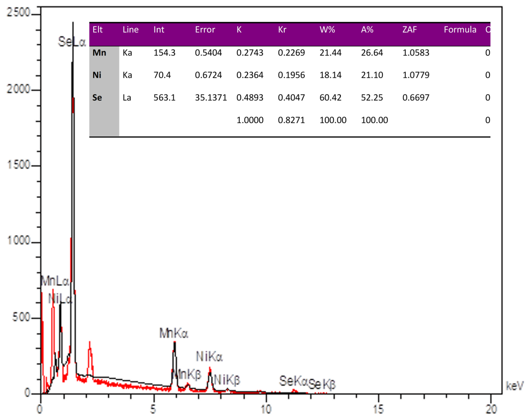

(a)

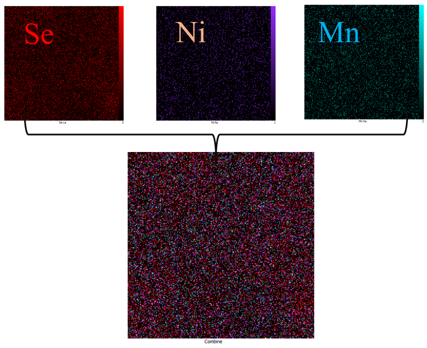

(b)

Figure S4: Energy-Dispersive X-ray Spectroscopy (EDS) analysis of the nickel-added MnSe nanostructures: (a) EDS spectrum of the sample synthesized with a nominal impurity concentration of 2.0 mmol Ni, confirming the successful incorporation of nickel into the host matrix. (b) Corresponding EDS elemental mapping of the 2.0 mmol Ni-added sample, illustrating the spatial distribution of the constituent elements across the synthesized microstructures.
